# Dexmedetomidine Preserves Activity of Neurons in Primary Somatosensory Cortex Compared to Propofol and Ketamine

**DOI:** 10.3390/brainsci12121720

**Published:** 2022-12-15

**Authors:** Mu-Chao Xia, Juan Guo, Yan Ding, Zi-Qi Shi, Fang Du, Kai Wang, Chang-Hong Miao, Chao Liang

**Affiliations:** 1 Department of Anesthesiology, Zhongshan Hospital, Fudan University, Shanghai 200032, China; 2 Shanghai Key Laboratory of Perioperative Stress and Protection, Shanghai 200032, China; 3 Institute of Neurology, Chinese Academy of Sciences, Shanghai 200031, China

**Keywords:** propofol, ketamine, dexmedetomidine, primary somatosensory cortex, neuron, anesthesia

## Abstract

General anesthesia has been shown to induce significant changes in the functional connectivity of the cerebral cortex. However, traditional methods such as electroencephalography (EEG) or functional magnetic resonance imaging (fMRI) lack the spatial resolution to study the effects of general anesthesia on individual cortical neurons. This study aimed to use high-resolution two-photon imaging, which can provide single-neuron resolution, to investigate the characteristics of consciousness under general anesthesia. We used C57BL/6J and Thy1-GCamp6s mice and found that at similar levels of sedation, as measured by EEG, dexmedetomidine did not significantly inhibit the spontaneous activity of neuronal somata in the S1 cortex, but preserved the frequency of calcium events in neuronal spines. In contrast, propofol and ketamine dramatically inhibited the spontaneous activity of both neuronal somata and spines. The S1 cortex still responded to whisker stimulation under dexmedetomidine anesthesia, but not under propofol or ketamine anesthesia. Our results suggest that dexmedetomidine anesthesia has unique neuronal properties associated with its ability to facilitate easy awakening in the clinic. These findings provide insights into the development of more effective strategies for monitoring consciousness during general anesthesia.

## 1. Introduction

There are many patients worldwide who receive general anesthesia each year. General anesthesia is a valuable medical tool that can safely induce reversible loss of consciousness. However, the exact mechanisms by which general anesthetics work remain a long-term problem for clinicians and researchers alike [1].

General anesthetics that act on different receptors can have different effects on the function of the cerebral cortex [2]. Recent studies have shown that the disruption of cortical connectivity is a key feature of general anesthesia [3,4]. Some functional magnetic resonance imaging (fMRI) studies have indicated that sevoflurane weakens the signal correlation between functionally related and specialized brain regions. As the concentration of anesthetics increases, resting-state networks have shown a gradual breakdown of intracortical and thalamic–cortical connections [5]. This is thought to be due to changes in the membrane potential of neurons that alter their ability to generate action potentials and transmit information [6]. It is important to determine whether this large-scale model of the cortex under anesthesia can be reflected at the level of individual neurons, and whether different anesthetics have different effects on neuronal firing patterns.

It is well known that general anesthesia affects the firing patterns of cortical neurons [7,8,9]. The primary somatosensory cortex (S1) plays a crucial role in processing tactile information from the body, including sensations such as touch, pressure, temperature, and pain [10]. This sensory information is then conveyed to the neocortex through neural pathways that connect to the spinal cord, brain stem, and thalamus, which are all closely associated with nerve circuits related to general anesthesia [11]. Neurons, particularly pyramidal neurons, are the key components of neural networks, and their main function is to conduct nerve impulses [12]. To this end, each neuron integrates thousands of synaptic inputs [6]. Despite the important role of the S1 cortex as a high-level center for processing sensory information, its activity under anesthesia has not been systematically studied.

In this study, we focused on three intravenous anesthetics: dexmedetomidine, propofol, and ketamine. These three anesthetics produce sedative effects through different receptors, with dexmedetomidine being a highly selective alpha-2 adrenergic receptor agonist, propofol being a gamma-aminobutyric acid type A (GABA-A) receptor agonist, and ketamine being an *N*-methyl-d-aspartate (NMDA) receptor antagonist [2]. Propofol is widely used in clinical practice, dexmedetomidine is an important adjuvant to other anesthetics that can reduce the required dosage, and ketamine has gained attention for its rapid antidepressant effects.

To begin, we calculated parameters of consciousness under EEG recording for the three anesthetics. We then used two-photon laser scanning microscopy to observe the spontaneous activity of neuronal somata and spines within the S1 cortex. Additionally, we combined wide-field imaging and whisker stimulation to record the evoked response of the neuronal population within the S1 cortex. Our goal was to investigate the cortical effects of different general anesthetics at high spatial resolution.

## 2. Materials and Methods

### 2.1. Animals and Experimental Grouping

In this study, we used adult male mice of the C57BL/6J strain (6–8 weeks of age at the start of the experiments) and Thy1-GCamp6s strain (No. 024275 Jackson Lab, Bar Harbor). The mice were group-housed (3–5 mice per cage) in a controlled environment with a 12-hour light/dark cycle and free access to food and water. The experimental procedures were approved by the Animal Care and Use Committee of the Institute of Neuroscience, Chinese Academy of Sciences (approval code NA-024-2019).

A total of 51 mice were used in the study. Of this total, 6 C57BL/6J mice were used for EEG recordings, and 36 C57BL/6J mice were used for two-photon imaging, with half of them used for soma imaging and the other half for spine imaging. There were 6 mice per drug group (dexmedetomidine, propofol, ketamine) for both soma and spine imaging. A total of 5 Thy1-GCamp6s mice were used for wide-field imaging, and data from the awake group were derived from self-control experiments. Finally, 4 C57BL/6J mice were used for the control group experiment.

The mice were randomly divided into experimental groups using a completely random design method.

### 2.2. Surgery

The mice were anesthetized with isoflurane in oxygen (at a concentration of 1–3%, delivered at a rate of 300 mL/min mixed with air) and placed in a stereotaxic apparatus. The body temperature was maintained at 37 °C using a heating pad, and the eyes were lubricated with an ophthalmic ointment. The skull was exposed by making an incision in the skin and sterilizing the area with ethanol and betadine. The center coordinate of the primary somatosensory cortex was determined using stereotaxic coordinates (anteroposterior (AP) = −1.5 mm, mediolateral (ML) = ±2.5 mm, dorsoventral (DV) = −0.3 mm). A 3 mm (5 mm for wide-field imaging) diameter craniotomy was performed over the right primary somatosensory cortex, leaving the dura intact. We then used a glass pipette to inject an AAV-hsyn-GCaMP6 virus solution (OBiO Technology Shanghai Corp. Ltd., Shanghai, China) into the brain, with a total volume of 100 nL (10 nL for spine imaging). The expression of the GCaMP protein in cortical cells is controlled by the synapsin promoter [13], so that only neurons (both excitatory and inhibitory) express the protein, but not glial cells. The skull window was covered with a glass coverslip (diameter 3 or 5 mm, thickness 0.15–0.17 mm) and sealed in place with dental cement. A metal headplate was then fixed to the skull using cyanoacrylate glue and dental cement to allow head fixation and imaging through the cranial window. The mice were allowed to recover for 3 weeks before the imaging experiments were started [9].

### 2.3. EEG Recording and Analysis

The mice were anesthetized with isoflurane as before and placed in a stereotaxic apparatus. Two EEG electrodes were surgically implanted in the frontal cortex (anteroposterior (AP) = +1.5 mm, mediolateral (ML) = ±0.5 mm, dorsoventral (DV) = −3.8 mm) and the midline cerebellum (reference electrode). The EEG signals were continuously recorded at a sampling frequency of 128 Hz using an acquisition card (PCI6221; National Instruments Corp., Austin, TX, USA) and Spike-hound software 3.0.5.

To analyze the EEG signals, we used the fft and bandpower functions in MATLAB 2021a. We calculated the power spectral density of theta (4–8 Hz) and delta (1–4 Hz) rhythms in the awake and anesthetized states, using data from 5-minute segments [14]. We then calculated the ratio of theta to delta (theta/delta) to normalize the data [15].

### 2.4. Electrical Stimulation Paradigm

To perform peripheral electrical stimulation, we used a pair of 30 G needle electrodes on each hind paw. Each pair of electrodes was connected to a stimulus isolator device (Model A365R, World Precision Instruments, Sarasota, FL, USA), which delivered a 2 s pulse (5 Hz, 0.5 mA, 5 V). For each mouse, the total acquisition time was 5 min, with each trial consisting of a 2 s stimulation followed by an 8 s interval. A total of 30 trials were performed per mouse.

### 2.5. Drug Application

All anesthetics were injected intraperitoneally, with the following doses: propofol (50 mL, 0.5 g, Fresenius Kabi, Linz, Austria) at 150 mg/kg, dexmedetomidine (2 mL, 200 μg, Hengrui, Lianyungang, China) at 100 μg/kg, and ketamine (50 mg/mL, Daiichi Sankyo, Tokyo, Japan) at 100 mg/kg. The anesthetics were quickly injected at the same time, and imaging was performed approximately 20 min after injection. Physiological saline and fat emulsion (500 mL:50 g, Kelun, Chengdu, China) were used as the vehicle controls.

The doses of the three drugs were chosen to ensure that the mice were unconscious and unresponsive [9,16,17]. During the data collection process, the mice underwent a 72 h washout period between experiments to ensure that any remaining drugs from the previous experiment were fully metabolized.

### 2.6. Two-Photon Calcium Imaging

We used a laser-scanning two-photon microscope to detect the GCaMP6s fluorescence signal. The signal was detected using a bandpass filter (No.525/50; Semrock, New York, USA) and GaAsP photomultiplier tubes (No.10770PB-40; Hamamatsu). We used a resonant galvanometer (Thorlabs, New Jersey, USA; 16 kHz line rate, bidirectional) for horizontal scanning, which was sealed in an optical window to reduce noise levels to below 30 dB. The entire microscope was enclosed in a double-walled sound-attenuation box to ensure that the internal noise level was below 30 dB during imaging. Images were obtained using the ScanImage software r3.0 (http://scanimage.org, accessed on 11 December 2022). Calcium transients were imaged in the cortex 200–300 μm beneath the pial surface, using 512 × 512 (soma) or 1024 × 1024 (spine) pixel images. We recorded the spontaneous neuronal activity in the mice for 5 min once they had reached a stable state of anesthesia, approximately 20 min after injection. The resolution of the dendritic spines was 0.3 μm per pixel, while the resolution of the cell bodies was 0.8 μm per pixel [9].

### 2.7. Wide-Field Fluorescence Microscopy Imaging and Whisker Stimulus

Wide-field calcium imaging was progressed using a customized tandem-lens epifluorescence macroscope with two optics lenses (85 mm f/1.8D objective, 50 mm f/1.4D tube lens, Nikon, Tokyo, Japan) placed in a face-to-face orientation. Excitation light passed from two LEDs, 470 nm (M470L3, Thorlabs, New Jersey, USA, with excitation filter FF02-447/60-25, Semrock, New York, USA) and delivered in the Koehler configuration through a dichroic mirror (FF495-Di03, Semrock, New York, USA) which was placed in the infinity-focused imaging path. The average power was about 0.05 mW/mm^2^, similar to that in other research. Images were captured using an emission filter (525/50-25/Semrock, New York, USA) and an sCMOS camera (pco.edge 5.5, PCO, Kelheim, Germany) at 60 Hz in a rolling shutter mode and binned on the fly 2 × 2 using the manufacturer software. This lens combination resulted in a resolution of about 20 μm per pixel. Excitation wavelengths were interleaved by a microcontroller (Teensy3.2) triggered by the camera rolling shutter exposure output. To avoid rolling shutter artifacts and crosstalk between 470 nm and 405 nm excitation frames, we restricted illumination to periods when all the lines being acquired corresponded to the same imaging frame [18]. During the whisker stimulation experiments, a rubber balloon was used to deliver the stimulus to the mice’s whiskers, as previously described. The stimulus was delivered continuously for 10 s starting at the 20th second of imaging, using 10 ms air pulses delivered at a frequency of 3–5 Hz. The mice were kept in a quiet environment during the experiments, with the use of a shading cloth to isolate them from ambient light. Each trial lasted for 1 min.

### 2.8. Data Acquisition and Analysis

Data collection was not performed blind to the conditions of the experiments, but the analysis was performed blind to the conditions of the experiments to minimize subjective bias in the analysis. Batch and automatic analyses were performed for the control and experimental groups, and all imaged data were analyzed using MATLAB 2021a and ImageJ software. Regions of interest (ROIs) were manually drawn using a customized MATLAB code, and the total response value of all pixels within an ROI was used as the response value for that ROI. The fluorescence intensity time series for each ROI was calculated, and the distribution of all fluorescence values was recorded simultaneously. The baseline fluorescence value (F0) was defined as the fluorescence value corresponding to the peak of the distribution. For each ROI, the relative change in fluorescence (ΔF/F0) was calculated as (F − F0)/F0 × 100%. When imaging cell bodies, a calcium event was defined as a ΔF/F0 value of at least 80%, and when imaging dendritic spines, a calcium event was defined as a ΔF/F0 value of at least 80% [19]. Then, we manually checked to ensure that each ROI signal matched, and signals with excessive errors were discarded. The data are represented as mean ± SD in all figures.

### 2.9. Statistical Analysis

For statistical analysis, we used an unpaired t-test for two independent groups where n ≥ 20 and data were normally distributed. For non-normally distributed data or when n < 20, we used the Wilcoxon rank-sum or Kruskal–Wallis tests for unpaired comparisons and the Wilcoxon signed-rank test for paired comparisons. Whenever necessary, we performed Bonferroni correction for multiple testing.

## 3. Results

### 3.1. Dexmedetomidine, Propofol, and Ketamine Could Induce the Same Depth of Sedation

**Figure 1:**
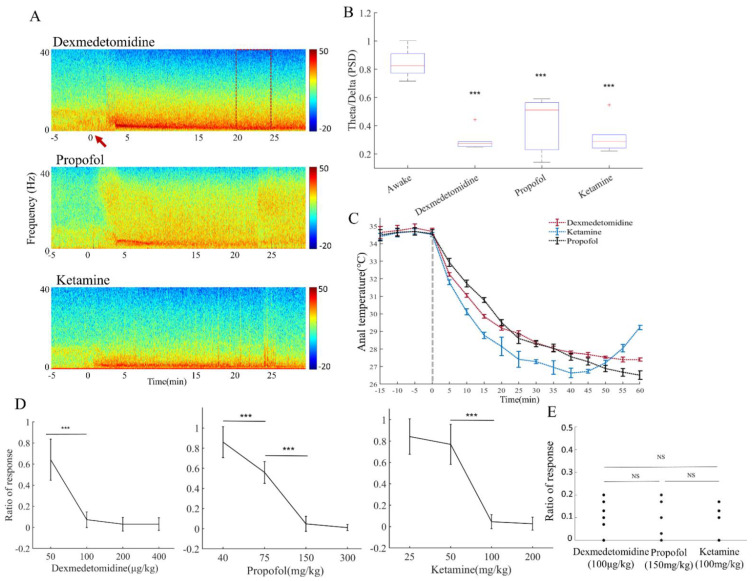
A similar sedative depth was induced by the three anesthetics. (**A**) Schematic diagram of the EEG of 100 μg/kg dexmedetomidine, 150 mg/kg propofol, and 100 mg/kg ketamine, with the red arrow indicating the injection time point and the red frame indicating the 5 min period selected for the follow-up experiment (20th to 25th minutes after injection). (**B**) Power spectral density statistics for 5 min of awake and 5 min of anesthesia, normalized to the ratio of theta (4–8 Hz) to delta (1–4 Hz) to show changes in EEG power. *** *p* < 0.001, n = 14 (awake), n = 6 (dexmedetomidine), n = 6 (propofol), n = 6 (ketamine), One-way analysis of variance. (**C**) Anal temperature curve of mice after injection (100 μg/kg dexmedetomidine, 150 mg/kg propofol, 100 mg/kg ketamine). (**D**) Dose gradients of an electrical stimulus applied to mice’s hind paws. *** *p* < 0.001, n = 12 per group (dexmedetomidine), n = 11 per group (propofol), n = 11 per group (ketamine), Chi-square analysis. (**E**) The response of mice to the electrical stimulus at the target dose. n = 12 (dexmedetomidine), n = 11 (propofol), n = 11 (ketamine), Chi-square analysis.

First, we performed EEG monitoring on mice to test the commonly used doses of dexmedetomidine (100 μg/kg), propofol (150 mg/kg), and ketamine (100 mg/kg). We recorded five minutes of the awake period as a baseline, then injected the drug intraperitoneally. We found that after the injection, the mice’s EEGs changed rapidly, with the time–frequency graphs of all three anesthetics showing a stable state of sedation at the 20th minute (Figure 1A). All three groups of mice showed a decrease in theta rhythm power and an increase in delta rhythm power [15] between the 20th and 25th minutes compared to wakefulness (Figure 1B) (mean difference (I-J) = 0.5395, *p* < 0.001 (dexmedetomidine); mean difference (I-J) = 0.4116, *p* < 0.001 (propofol); mean difference (I-J) = 0.5158, *p* < 0.001 (ketamine)). Body temperature plots at these doses also suggest that mice were at similar levels of hypothermia (Figure 1C). We then tested the response of mice to hind paw electrical stimulation under different concentrations of anesthetics [20]. We defined that the ratio of average response of mice less than 20% is an unresponsive state. Based on this standard, we set up a dose gradient to stimulate the mice. The results showed that dexmedetomidine (100 μg/kg), propofol (150 mg/kg), and ketamine (100 mg/kg) could significantly reduce the mice’s response to external stimulus (Figure 1D) (χ^2^ = 54, *p* < 0.001 (dexmedetomidine), χ^2^ = 66, *p* < 0.001; χ^2^ = 22, *p* < 0.001 (propofol), χ^2^ = 33.9176, *p* < 0.001 (ketamine)). These results confirm that the selected doses of the three anesthetics produce a similar level of sedation in the mice, as indicated by both EEG and behavioral measures. There was also no significant difference in the proportion of responses between the three doses (Figure 1E).

### 3.2. Preserved Neuronal Activity in the S1 with the Administration of Dexmedetomidine Rather than Propofol and Ketamine

**Figure 2:**
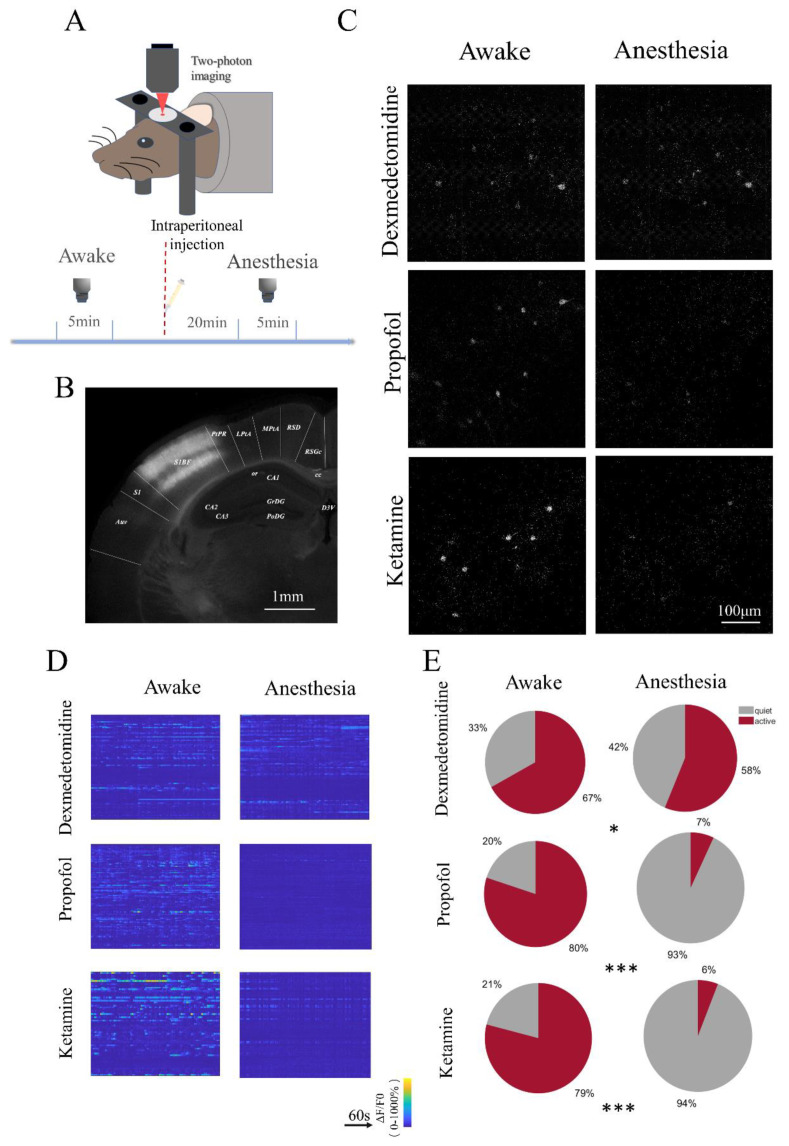
Ratio of active neurons under the three anesthetics of S1 neurons. (**A**) Two-photon laser scanning microscopy (2PLSM) was used to image spontaneous S1 neuron calcium signals in awake and anesthetized head-restrained mice. Each mouse was scanned for 5 min under awake and anesthesia state. (**B**) Coronal histological section, showing the extent of AAV-mediated expression of GCamp6s in the mouse primary somatosensory cortex. (**C**) The field of view of the S1 population neurons under a two-photon microscope showing the awake and dexmedetomidine, propofol, and ketamine anesthesia states. (**D**) Calcium signal firing of all neurons before and after anesthesia in the same field of view of the three anesthetics within 5 min. The vertical axis represents the order of neurons. (**E**) Pie chart of the ratio of active cells to quiet cells in the awake state and the anesthetic state of the three anesthetics (active cell: calcium events ≥1 in 5 min). *** *p* < 0.001, * *p* = 0.040 (n = 94 (quiet), n = 191 (active), awake; n = 102 (quiet), and n = 143 (active), dexmedetomidine; n = 29 (quiet), n = 113 (active), awake; n = 124 (quiet), and n = 10 (active), propofol; n = 37 (quiet), n = 142 (active), awake; n = 186 (quiet), and n = 12 (active), ketamine, Chi-square analysis).

In order to observe the response of S1 neurons under two-photon laser scanning microscopy (2PLSM) according to the established anesthesia plan (Figure 2A), we injected a calcium indicator into S1 to make neurons in almost the entire cortex expressing fluorescence (Figure 2B). In the field of view of 2PLSM, we found that neuronal activity did not change significantly after dexmedetomidine injection. However, the neuronal activity in mice anesthetized with propofol or ketamine was completely inhibited (Figure 2C,D). Although the proportion of active cells (calcium events ≥ 1 in 5 min) decreased after the administration of three anesthetics (χ^2^ = 4.2299, *p* = 0.040 (dexmedetomidine); χ^2^ = 145.129, *p* < 0.001 (propofol); χ^2^ = 208.869, *p* < 0.001 (ketamine)), the percentage of active cells in the dexmedetomidine group (58%) was much higher than that in the other two groups (7%, 6%) (Figure 2E). The drug carriers, physiological saline and fat emulsion, did not cause changes in S1 neuron activity (Figure S1).

### 3.3. Amplitude and Frequency of Calcium Events in the S1 Neuronal Somata Maintained under Dexmedetomidine Rather than Propofol and Ketamine Anesthesia

**Figure 3:**
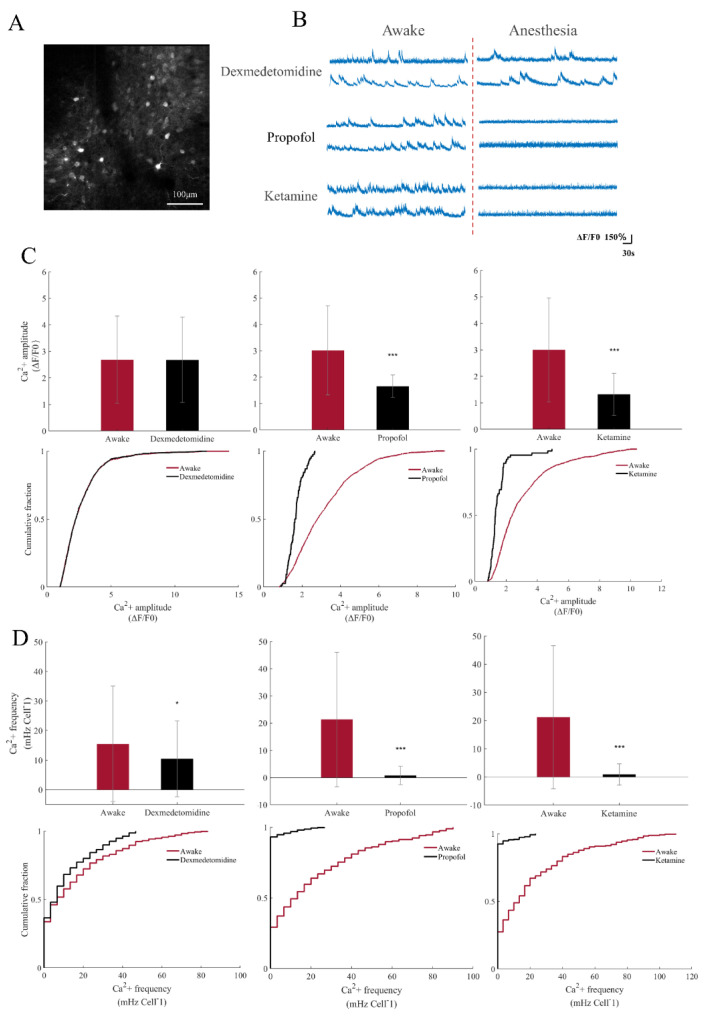
Analysis of calcium signal of soma before and after anesthesia with three anesthetics of the S1 neurons. (**A**) Gcamp6s-expressing neurons of S1 in C57 mice. (**B**) ΔF/F0 traces of spontaneous calcium signal illustrate the effects of anesthetic induction (dexmedetomidine 100 μg/kg, propofol 150 mg/kg, and ketamine 100 mg/kg) on neuronal signaling. (**C**) Mean calcium event amplitude induced by anesthetics in awake and anesthetized mice. *** *p* < 0.001 (n = 1922 (awake), n = 1110 (dexmedetomidine) calcium events from 6 animals, *p* = 0.9179, T-test analysis; n = 1368 (awake), and n = 65 (propofol) calcium events from 6 animals, corrected T-test analysis; n = 2169 (awake), and n = 76 (ketamine) calcium events from 6 animals, corrected T-test analysis) (top). Cumulative function distribution diagram of S1 neuron calcium event amplitude under awake and anesthetic states of three drugs (bottom). (**D**) Mean calcium event frequency induced by anesthetics in awake and anesthetized mice. *** *p* < 0.001, * *p* < 0.05 (n = 445 (awake), n = 375 (dexmedetomidine) cells from 6 animals, Wilcoxon rank-sum test; n = 237 (awake), and n = 264 (propofol) cells from 6 animals, Wilcoxon rank-sum test; n = 369 (awake), and n = 361 (ketamine) cells from 6 animals, Wilcoxon rank-sum test) (top). Cumulative function distribution diagram of the S1 neuron calcium event frequency under awake and anesthetic states of three drugs (bottom).

We then analyzed the activity of each neuronal somata in the whole field (Figure 3A). We defined the signal as a calcium event of a neuron when ΔF/F0 ≥ 80%. There were differences in neuronal signals before and after anesthesia with the three anesthetics (Figure 3B). The magnitude of the calcium signal of each calcium event in these cells and the number of calcium events per unit time were measured. The results showed that compared to the awake state, the amplitude of the calcium event had no significant changes under dexmedetomidine anesthesia (t = 0.1031, *p* = 0.9179), while the frequency of the calcium event decreased significantly (Z = 2.5170, *p* = 0.0118). However, under propofol and ketamine anesthesia, both calcium signal amplitudes of calcium events decreased significantly (t = 9.1014, *p* < 0.001 (propofol); t = 8.3654, *p* < 0.01 (ketamine)), and the frequency of calcium events approached zero (Z = 14.8723, *p* < 0.001 (propofol); Z = 17.5803, *p* < 0.001 (ketamine)), results which were significantly different from those caused by dexmedetomidine (Figure 3C,D).

### 3.4. Frequency of Calcium Events in the S1 Neuronal Spine under Dexmedetomidine Anesthesia Retained Same as Awake State

**Figure 4:**
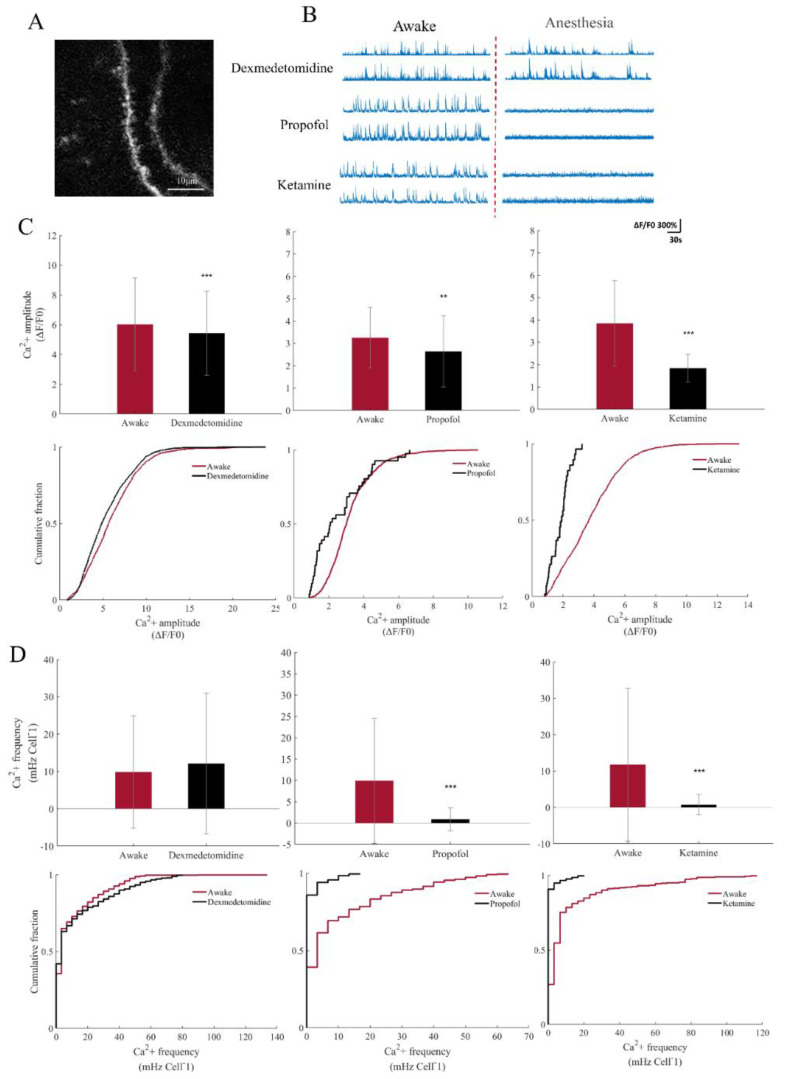
Analysis of calcium signal of the spine before and after anesthesia with three anesthetics of the S1 neurons. (**A**) Gcamp6s-expressing spines of S1 in C57 mice. (**B**) ΔF/F0 traces of spontaneous calcium signal illustrate the effects of anesthetic induction (dexmedetomidine 100 μg/kg, propofol 150 mg/kg, and ketamine 100 mg/kg) on spines’ signaling. (**C**) Mean calcium event amplitude induced by anesthetics in awake and anesthetized mice. *** *p* < 0.001, ** *p* < 0.01 (n = 1641 (awake), n = 1921 (dexmedetomidine) calcium events from 6 animals, corrected T-test analysis; n = 1033 (awake), and n = 57 (propofol) calcium events from 6 animals, corrected T-test analysis; n = 973 (awake), and n = 41 (ketamine) calcium events from 6 animals, T-test analysis) (top). Cumulative function distribution diagram of S1 neuron calcium event amplitude under awake and anesthetic states of three drugs (bottom). (**D**) Mean calcium event frequency of the spine induced by anesthetics in awake and anesthetized mice. *** *p* < 0.001, ** *p* < 0.01 (n = 567 (awake), n = 536 (dexmedetomidine) cells from 6 animals, Wilcoxon rank-sum test; n = 302 (awake), and n = 289 (propofol) cells from 6 animals, Wilcoxon rank-sum test; n = 329 (awake), and n = 184 (ketamine) cells from 6 animals, Wilcoxon rank-sum test) (top). Cumulative function distribution diagram of S1 spine calcium event frequency under awake and anesthetic states of three drugs (bottom).

Neuronal synapses are an important cellular substructure for electrical signal transduction, and the spine receives input from other neurons. More insight into the cortical site disrupted by general anesthesia can be obtained by focusing on the calcium signal of the spine [21]. We adjusted the resolution to photograph the synapses in mice anesthetized with three different drugs (Figure 4A), and still defined the signal as a calcium event of the spine when ΔF/F0 ≥ 80%. Similarly, there were differences in spinal signals before and after anesthesia with the three anesthetics (Figure 4B). The results indicated that the amplitude of the calcium signal decreased significantly in mice anesthetized with three drugs (t = 5.9276, *p* < 0.001 (dexmedetomidine); t = 2.8902, *p* = 0.0053 (propofol); t = 10.6786, *p* < 0.001 (ketamine)). The frequency of the calcium signal showed no statistical difference, but the mean showed an upward trend in dexmedetomidine-anesthetized mice (Z = 0.3374, *p* = 0.7358), while it decreased in propofol- and ketamine-anesthetized mice (Z = 15.1831, *p* < 0.001 (propofol); Z = 10.3212, *p* < 0.001 (ketamine)) (Figure 4C,D).

### 3.5. The S1 Cortex Maintained Its Response to Whisker Stimulus under Dexmedetomidine Anesthesia Rather than Propofol and Ketamine Anesthesia

**Figure 5:**
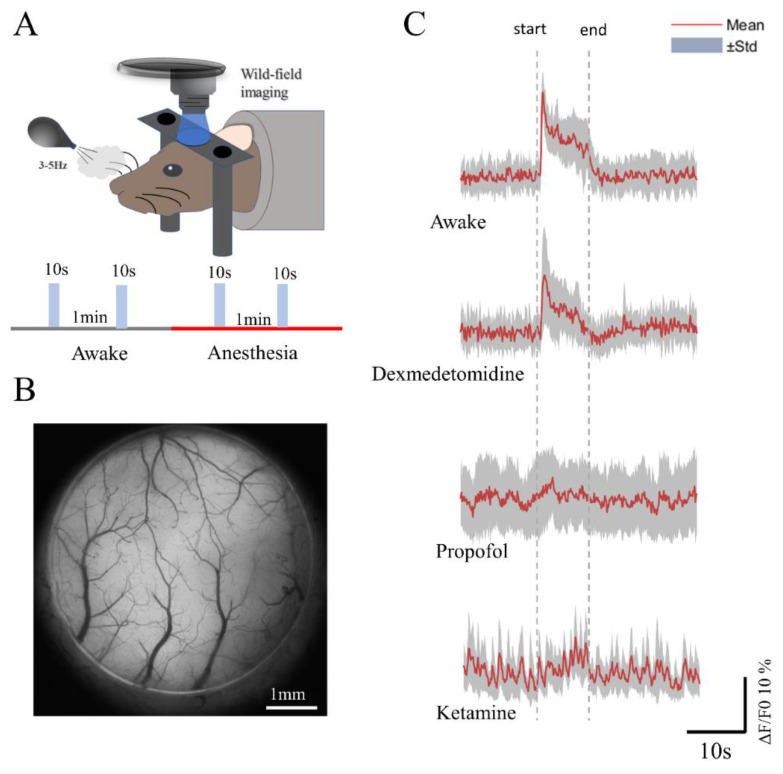
Results of whisker-blowing stimulus in mice under awake and anesthesia states with three anesthetics. (**A**) Schematic diagram of whisker-blowing stimulus for head-fixed mice (Thy1-Gcamp6s). (**B**) The wide-field imaging field of view taken during blowing stimulus for subsequent analysis. (**C**) ΔF/F0 traces of calcium signal of S1 cortex when in awake and anesthesia states (n = 40 (awake), n = 39 (dexmedetomidine), n = 40 (propofol), and n = 40 (ketamine) trials from 5 animals). The red dotted line represents the start and end times of the stimulus.

Finally, to further capture the cortical response to external stimuli rather than spontaneous activity, we observed the response of the entire S1 cortex to whisker stimulus in mice anesthetized with the three drugs (Figure 5A). A 5 mm skull window was constructed for wide-field imaging (Figure 5B). We used Thy1-Gcamp6s mice, whose fluorescence was expressed throughout the cortex. Our results showed that in the awake state, the response of the mice to whisker-blowing stimulus was characterized by a rapid rise in the calcium signal followed by a slow decline. During dexmedetomidine anesthesia, a significant increase in the calcium signal was also observed; however, the duration of the plateau was shorter. While mice anesthetized with propofol and ketamine showed no obvious response to whisker-blowing stimulus, the S1 cortex showed regular calcium signal oscillations after ketamine anesthesia (Figure 5C).

## 4. Discussion

The main purpose of this study was to explore the response of cortical neurons in the S1 region to dexmedetomidine, propofol, and ketamine at the same level of sedation. We found that there was no significant difference in soma activity between dexmedetomidine-induced sedation and the awake state, while dexmedetomidine was found to preserve the frequency of synaptic activity. Propofol and ketamine completely inhibited neuronal activity in the somata and spines. Furthermore, mice anesthetized with dexmedetomidine retained an active response to whisker stimuli, which disappeared in mice anesthetized with propofol and ketamine.

Cortical connectedness is considered to be independent of the level of consciousness [22]. We investigated the effects of different anesthetics on cortical connectedness at a level of unconsciousness and unresponsiveness. At the level of sedation we set, dexmedetomidine induces a decrease in the calcium events of neuronal somata within the S1 cortex but preserves the frequency of calcium events in the neuronal spine. The distinct pattern of the neuronal soma and spine within the S1 cortex indicates a unique information integration mechanism under dexmedetomidine anesthesia. Specifically, the neuron in the S1 cortex still retains the ability to receive the information input, while not all of the input can trigger the neuronal calcium activity [23]. Compared to the awake state, the amplitude of the calcium activity in the neuronal spine is lower in the dexmedetomidine anesthetized state. We speculate that the paradoxical effects of dexmedetomidine on the amplitude and frequency of the neuronal spine may be due to an increased proportion of neurons with low-amplitude calcium activity. The increased low-amplitude calcium activity of spines under dexmedetomidine probably cannot trigger neuronal soma activity. This suggests that the ability of the spines of S1 neurons to receive sensory input is maintained, but the fact that the proportion of high-amplitude activity in the resting state is less means that it is difficult to cause large-scale neuronal network changes that are sufficient to induce spontaneous behavioral performance in mice. In addition, we observed the effect of dexmedetomidine on the average neuronal activity in the S1 cortex in response to whisker-stimulus and found a preserved evoked activity in the neuronal population in S1. This principle of the redistribution of spontaneous activity in the neuronal spine preserved spontaneous activity in the neuronal soma and preserved evoked activity in the neuronal population, which explains the dexmedetomidine-induced unique sedation pattern—easy-to-awaken—and indicates the preserved functional connectivity of the cortex under dexmedetomidine-induced sedation [24]. Our study provides an experimental paradigm and specific target for research focused on the mechanism of dexmedetomidine.

It has been suggested that the locus coeruleus–norepinephrine (LC-NE) system plays a key role in sensory signal processing to facilitate information integration such as decision making and motor response [25,26]. Most studies generally claim that LC-NE activation facilitates the representation of sensory signals by inhibiting spontaneous neuronal activity more than sensory-evoked response, effectively enhancing the signal-to-noise ratio (SNR) at the population level [27,28]. In addition, the ventral tegmental area-dopamine (VTA-DA) system has also been reported as an important target involved in dexmedetomidine-induced sedation [29]. Recently, a study reported that dexmedetomidine activates dopamine neurons in the ventral tegmental area and increases dopamine concentrations in the related forebrain projection areas [24], and they thought that dexmedetomidine-induced activation of the DA system attenuates the depth of sedation. Our result of neuronal spine under dexmedetomidine anesthesia is indeed in line with the presynaptic target of dexmedetomidine. The redistribution of calcium events in the neuronal spine based on the amplitude and the decreased frequency of calcium events in the neuronal soma indicates a presynaptic mechanism involvement and a decreased signal-to-noise ratio of spontaneous neuronal activity [30]. Our results suggest that the activation of dexmedetomidine at the midbrain VTA may also be reflected in the downstream S1 brain area. Further research is needed to understand the roles of presynaptic alpha2-adrenergic receptors, LC-NE, VTA-DA, and other neurotransmitter systems in the regulation of neuronal activity.

The 2PLSM results indicate that propofol almost completely inhibits spontaneous activity in the neuronal soma and spine, as well as the evoked activity of the neuronal population. However, the effect of propofol on cortical activity is debatable. The fMRI results show that during the period of non-response caused by propofol, thalamic functional connectivity is not reduced for unresponsive states within lower-order (auditory, sensorimotor, and visual) networks [31]. However, our previous research found that anesthetic doses of propofol can inhibit calcium transients and neuronal activity in the primary auditory cortex of mice [9]. Taken together with the new evidence from the S1 cortex, we propose that propofol causes the S1 cortex to become unresponsive to external information input. Differences between results from single-neuron brain regions may be due to the scale of observation. This kind of inhibition of spontaneous and evoked activity by propofol may be due to direct action on GABAA receptors, which are mostly distributed in the synaptic cleft of cortical neurons.

Similarly, ketamine suppressed spontaneous activity and evoked activity of the S1 cortex. The behavioral effects of ketamine depend on its dose. Low doses of ketamine can have antidepressant effects, while high doses can cause anesthesia [19]. However, there is still a lack of evidence regarding the effects of anesthetic doses of ketamine on the characterization of cortical neurons. Our results confirm that in the S1 cortex, anesthetic doses of ketamine lead to the general inhibition of neurons. As the dose of ketamine increases, the behavior of the neural network changes. Low-dose ketamine selectively inhibits NMDA receptors located on GABAergic interneurons [32,33]. Under anesthetic doses, ketamine may directly act on the NMDA receptors on the postsynaptic membrane of excitatory neurons, leading to its inhibitory effect on neurons [34].

In addition, we found a different pattern of stimulus-related neuronal population response in the S1 cortex under dexmedetomidine and awake states. Similar to the awake state, dexmedetomidine-anesthetized mice were still able to respond to whisker stimulus. Notably, compared to the continuous plateau response in the awake state, the calcium signal under dexmedetomidine anesthesia rapidly declined. Specifically, continuous whisker stimulation in awake mice was accompanied by a rapidly rising peak followed by a plateau that lasted for the majority of the stimulus period. This continuous signal was interrupted under dexmedetomidine sedation, with a rapid decline appearing after the initial increase in calcium signal activity. We speculate that dexmedetomidine may upregulate the threshold for response to environmental stimuli and disrupt the reinforcement mechanism for external stimuli [35]. Propofol and ketamine completely blocked the response of the local module of the S1 cortex, rendering it unresponsive to stimuli.

Our study has several limitations. First, while we ensured that the mice were unconscious and unresponsive, we did not record their vital signs simultaneously. Second, we only analyzed the unique effects of dexmedetomidine, propofol, and ketamine on the S1 cortex at the single-cell level, and did not investigate their effects on other cortical brain areas. Third, we did not differentiate between different types of neurons, and mainly observed neurons in the 2/3 layer of the S1 cortex. Different types of neurons may have different roles in the process of anesthesia. Finally, we did not further explore the relationship between the cortex and subcortex. The deep nuclei may play a more important role in anesthesia, and the behavior of the S1 cortex may be influenced by the activity of the deep nuclei.

## 5. Conclusions

Our study provides evidence for the unique effects of dexmedetomidine on the S1 cortex during anesthesia. Dexmedetomidine was shown to preserve neuronal activity in the neuronal spine, indicating that the ability of neurons to receive sensory input was maintained. This finding, combined with the preserved evoked activity in the neuronal population, suggests that dexmedetomidine-induced sedation is characterized by easy awakening and preserved functional connectivity in the cortex. The mechanisms underlying these effects are not yet fully understood and require further research. Additionally, our results demonstrate the inhibitory effects of propofol and ketamine on individual neurons in the S1 cortex, leading to the disruption of cortical functional connectivity. These findings have important implications for understanding the mechanisms of general anesthesia and may provide new insights into the depth of anesthesia monitoring.

## Data Availability

All data reported in this paper will be shared by the corresponding author upon request.

## Supplementary Materials

The following supporting information can be downloaded at: https://www.mdpi.com/article/10.3390/brainsci12121720/s1. Figure S1: Physiological saline and fat emulsion did not alter neuronal activity.
