# Supplementary material for: Dexmedetomidine Preserves Activity of Neurons in Primary Somatosensory Cortex Compared to Propofol and Ketamine
Source: Brain Sci. 2022 Dec 15;12(12):1720. doi: 10.3390/brainsci12121720 (PMC9775739; doi:10.3390/brainsci12121720)
Supplement: Supplementary file 1 [file brainsci-12-01720-s001.zip › brainsci-1974770-supplementary.pdf]

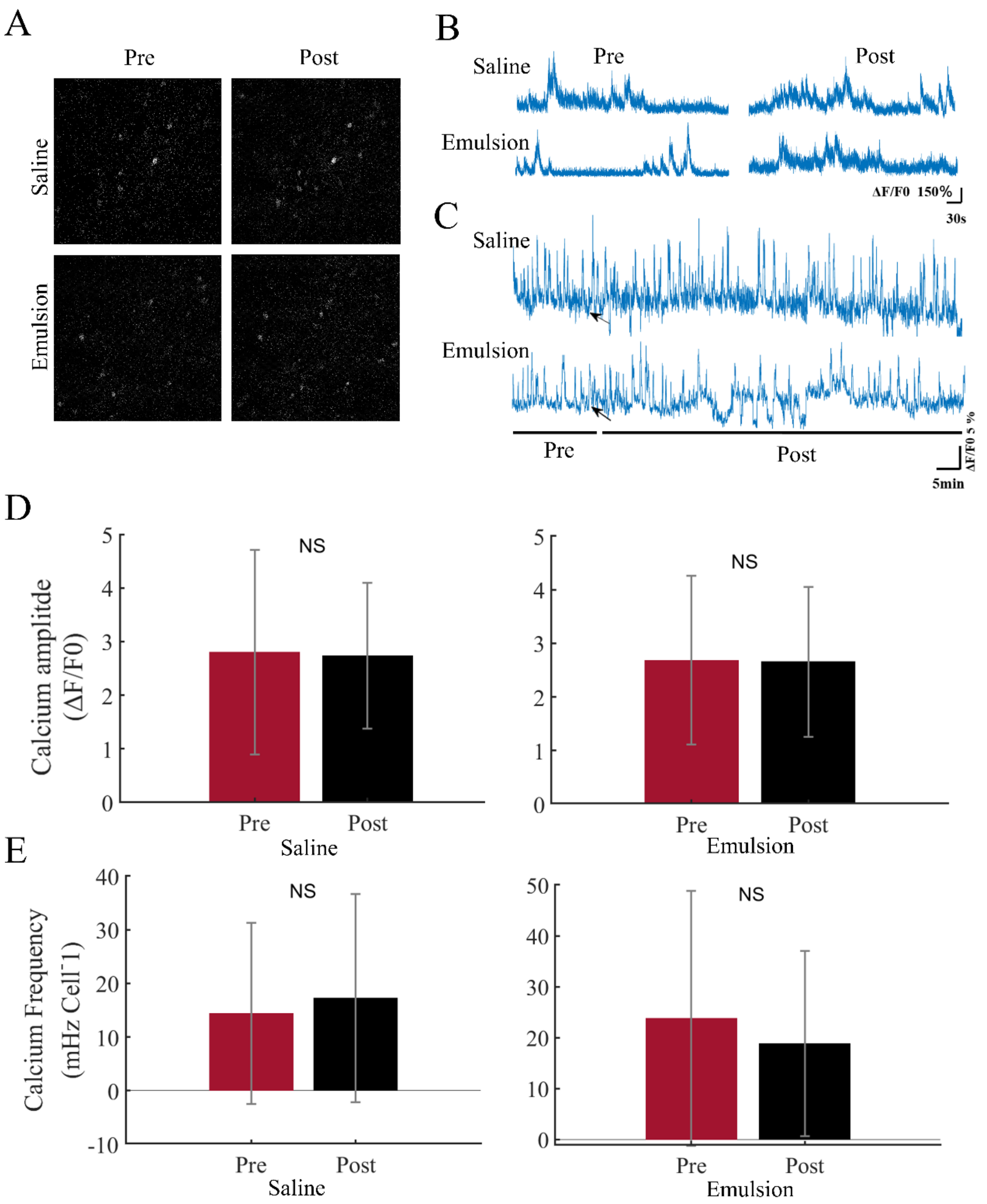

Figure S1. Physiological saline and fat emulsion did not alter neuronal activity. (**A**). Neuronal activities in the field of view of a two-photon microscope before and after injections of physiological saline and fat emulsion. (**B**). Examples of 5-minute segments of calcium signal curves for individual neurons before and after injections of physiological saline and fat emulsion. (**C**). Examples of 30-minute segments of calcium signal curves for the primary somatosensory cortex before and after injections of physiological saline and fat emulsion, with the arrow indicating the time of injection. (**D**). Average values of the amplitude of calcium events of S1 neurons before and after injections of physiological saline and fat emulsion. P = 0.51 P = 0.74 [n = 431 (Pre), n = 548 (Post) calcium events from 4 animals, Saline; n = 601 (Pre), n = 629 (Post) calcium events from 4 animals, Emulsion, T-test analysis] (**E**). Average values of the frequency of calcium events of S1 neurons before and after injections of physiological saline and fat emulsion. P = 0.2511 P = 0.1825 [n = 118 (Pre), n = 132 (Post) cells from 4 animals, Saline; n = 101 (Pre), n = 143 (Post) cells from 4 animals, Emulsion, Wilcoxon rank-sum test]
